# Treatment of Miller Class I Gingival Recession with Using Nonpedicle Adipose Tissue after Bichectomy Surgical Technique: A Case Report

**DOI:** 10.1155/2019/1049453

**Published:** 2019-12-30

**Authors:** Carmen Lucia Mueller Storrer, Leonardo Luiz Muller, Janes Francio Pissaia, Carla Frehner Andrade, Claudia Roberta Tenório Trevisani, Tatiana Miranda Deliberador

**Affiliations:** School of Health Science, Universidade Positivo, Curitiba, Paraná, Brazil

## Abstract

Gingival recession is an oral health problem that affects a large part of the population. Several treatments are suggested in the current literature; among them is the use of buccal fat pad grafting. The objective of this case report is to describe the treatment of a Miller Class I gingival recession using a nonpedicled buccal fat pad graft immediately after performing the surgery for buccal fat pad removal (bichectomy technique). First, bilateral surgical removal of the buccal fat pad was performed with the main objective of eliminating oral mucosa biting. The recipient site was prepared to receive a portion of the fat pad that was cut and macerated in a size that was sufficient to cover the recession. The patient was followed up at 15, 30, 60, and 365 days postsurgery, and the results showed an elimination of the oral mucosa biting and complete coverage of the gingival recession. It was concluded that the nonpedicled buccal fat pad graft is another option for the treatment of Miller Class I recessions.

## 1. Introduction

Gingival recession is the apical displacement of the gingival tissue in relation to the cementoenamel junction resulting in the root surface being exposed to the oral environment [1]. This exposure may lead to dentin sensitivity, pain, risk of root caries, abrasion, and erosion lesions, in addition to not being esthetic. Its etiology is associated with several factors such as biofilm accumulation, chemical and mechanical trauma, orthodontic treatment, quantity and quality of keratinized gingiva, and occlusal trauma [2–4].

Success in the treatment of gingival recessions is directly related to its severity. In Miller Class I and Class II recessions, there is no interproximal tissue loss; thus, complete root coverage is feasible [5].

Different surgical techniques such as free gingival graft, flap repositioning, and connective tissue graft have been used in the treatment of gingival recessions. Treatments with pedicled buccal fat pad grafts are also described in the literature with satisfactory results regarding root coverage, clinical attachment level, and keratinized tissue gain [6, 7]. However, the use of nonpedicled buccal fat pad grafts is still poorly described [8].

Currently, the surgical technique of buccal fat pad removal/bichectomy technique (removal of part of the buccal adipose tissue) has been frequently performed in dentistry with the main objective of eliminating oral mucosa biting. In a systematic review of 8 of the 220 articles found, it was concluded that the bichectomy technique has an initial favorable outcome regarding facial aesthetics with a low rate of complications, but there is a lack of randomized clinical trials to evaluate long-term outcomes [9].

The objective of this case report is to describe the treatment of a Miller Class I gingival recession using a nonpedicled buccal fat pad graft immediately after performing the surgery for buccal fat pad removal.

## 2. Case History

Prior to the surgical intervention, the patient was informed about the surgical procedure and signed an ethical consent form.

**Figure 1:**
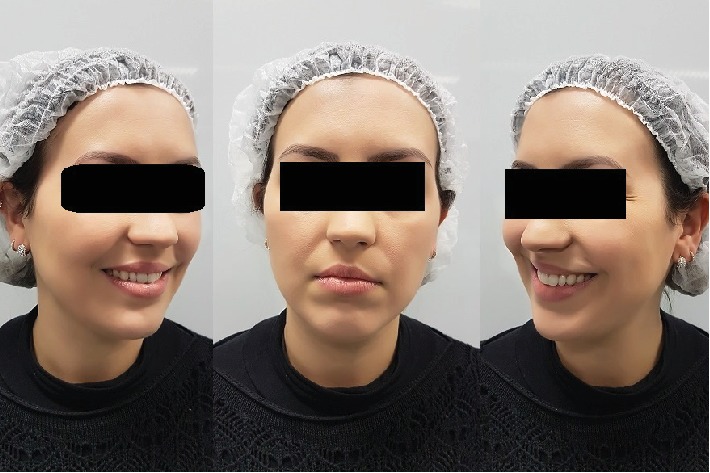
The clinical exam confirmed the face rounded and the indication for Bichectomy technique.

**Figure 2:**
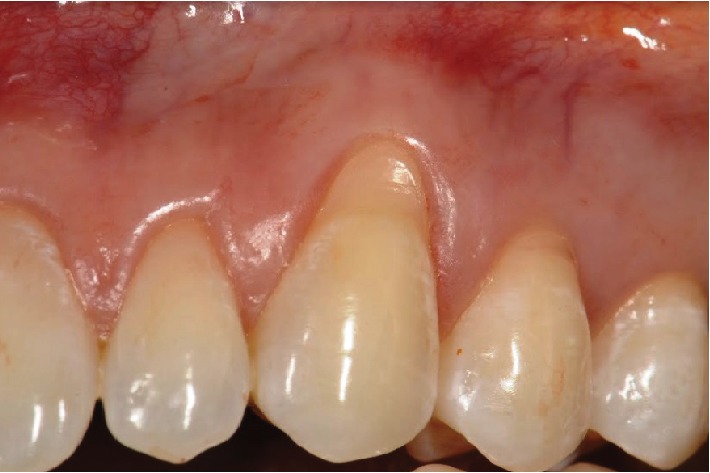
Miller Class I gingival recession on the maxillary left canine (recipient site).

The patient, a young female, presented to the Dentistry Clinic reporting a lack of gingival symmetry between the maxillary left and right canines and with the desire to eliminate oral mucosa biting. The clinical exam confirmed the face rounded (Figure 1) and cheek biting and revealed a Miller Class I gingival recession on the maxillary left canine (Figure 2). The removal of the Bichat fat pad was suggested to the patient with the objective of eliminating the oral mucosa biting and using the tissue for the root coverage.

**Figure 3:**
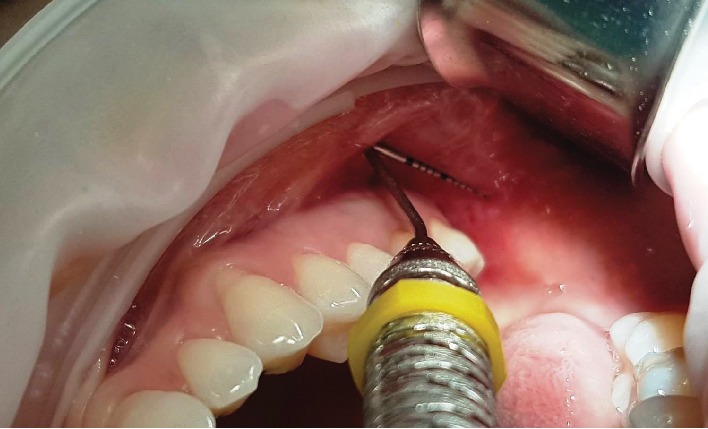
The incision location in donor site: vestibule fundus, at the distal caries of the second molar with a 30 mm distance from the vestibule and above the parotid duct.

**Figure 4:**
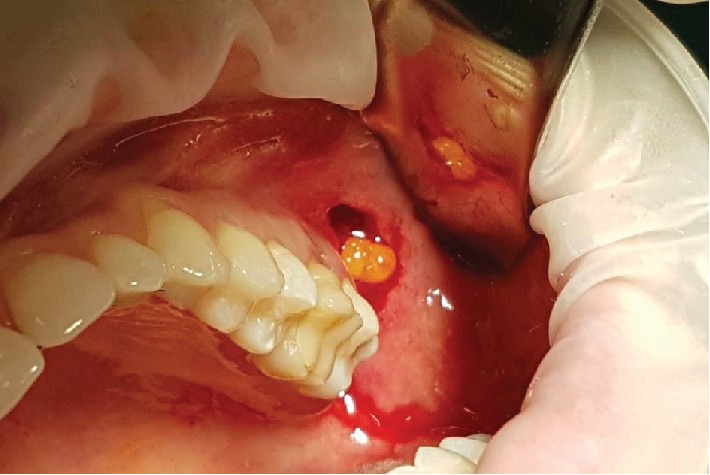
Extension of the incision: from the distal caries of the second molar to the mesial caries of the first molar.

**Figure 5:**
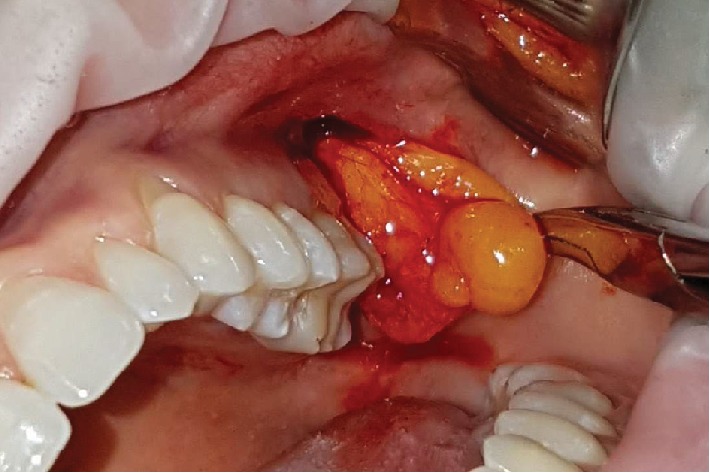
Removal of the buccal extension of the Bichat ball.

**Figure 6:**
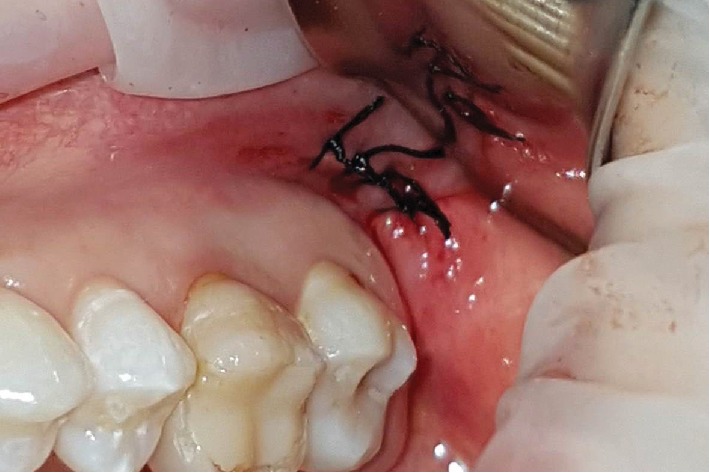
Simple suture in donor site with 4.0 silk thread.

The surgical procedure was initiated by harvesting the buccal fat pad. The incision location is at the vestibule fundus, at the distal caries of the second molar with a 30 mm distance from the vestibule and above the Parotid duct (Figure 3). The incision is made, initially, by perforating the alveolar mucosa, buccinator muscle, and connective tissue capsule that surrounds the Bichat buccal fat pad. Then, the incision is extended about 1.5 cm until it reaches the mesial caries of the first molar (Figure 4). A curved hemostat was used to enlarge the tissues. The buccal extension of the Bichat ball is completely removed by careful circular movements (Figure 5). 4 ml of buccal fat pad was removed. The donor site was immediately sutured with a 4.0 silk thread (braided black silk, Technofio, Goiania, GO, Brazil) with a simple suture (Figure 6).

**Figure 7:**
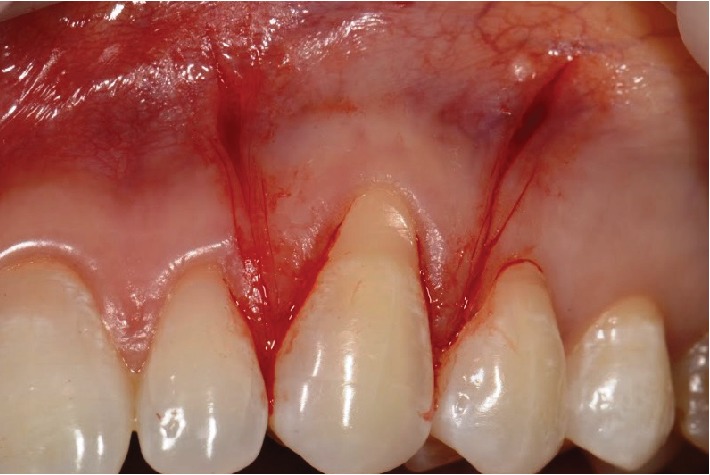
Preparation of the recipient site: with an intrasulcular incision and two diverging vertical incisions up to the alveolar mucosa.

**Figure 8:**
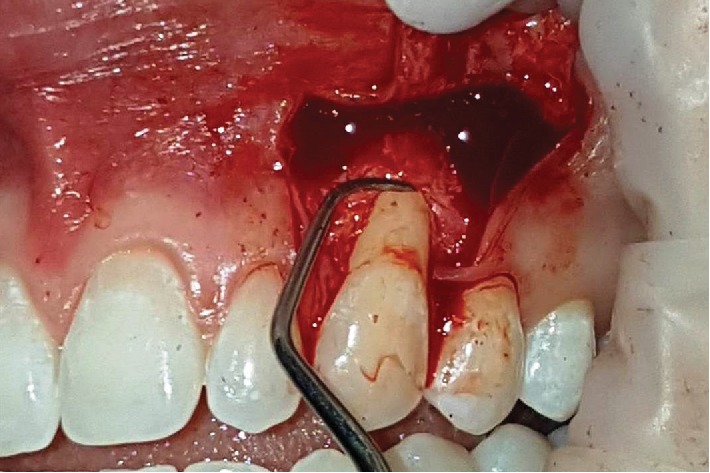
Scaled and planned with 5-6 Gracey curettes in canine root prior to receiving graft of adipose tissue.

**Figure 9:**
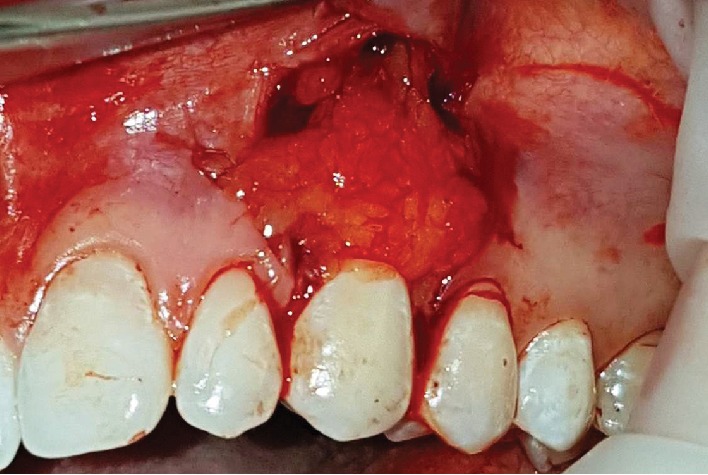
Macerated portion of the buccal fat pad cover of the recipient site.

**Figure 10:**
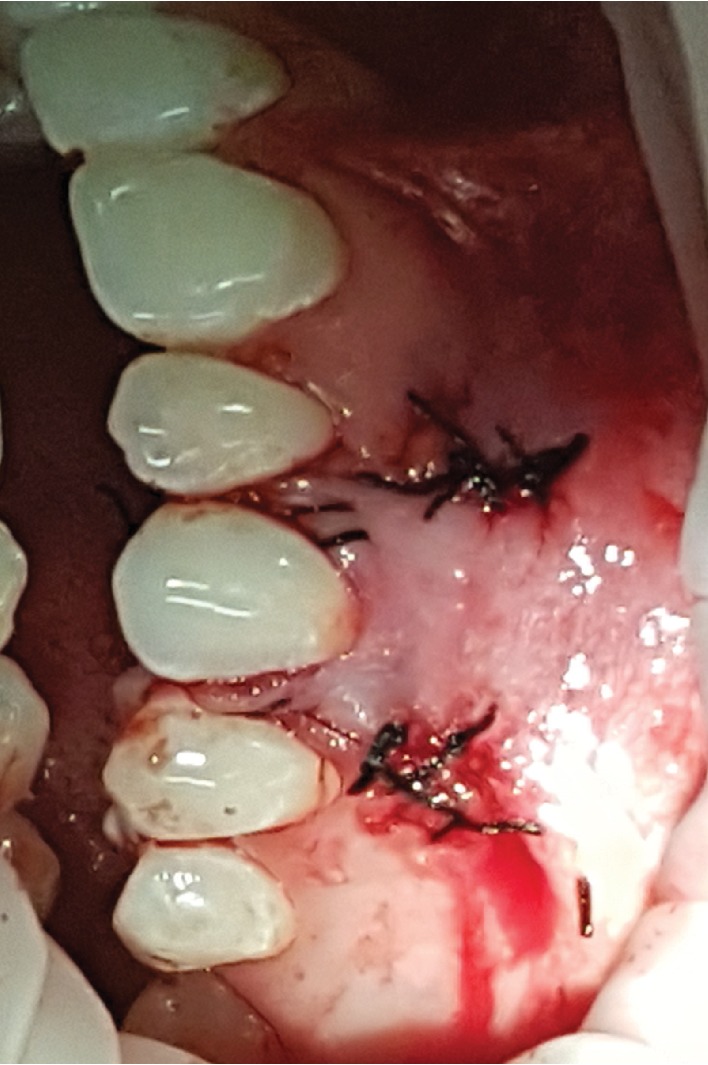
Flap coronally positioned and anchored with simple and suspension sutures.

The preparation of the recipient site (gingival recession on the maxillary left canine) was performed with an intrasulcular incision and two divergent vertical incisions toward the vestibule bottom (Figure 7). A partial thickness flap was then raised, and a periosteal releasing incision was made so the flap could be loosened and slid to cover the graft. The canine root was then scaled and planned with 5-6 Gracey curettes (Figure 8) (Hu-Friedy, Chicago, IL, USA) and irrigated with sterile saline solution. A portion of the buccal fat pad was cut into a size that was sufficient to cover the recipient site, its surface was macerated with a scalpel blade, and the graft was positioned (Figure 9). The graft was stabilized with an X suture using a 5.0 resorbable suture thread (polyglycolic acid suture, Technofio, Goiania, GO, Brazil), and the flap was then coronally positioned and anchored with simple and suspension sutures (Figure 10).

**Figure 11:**
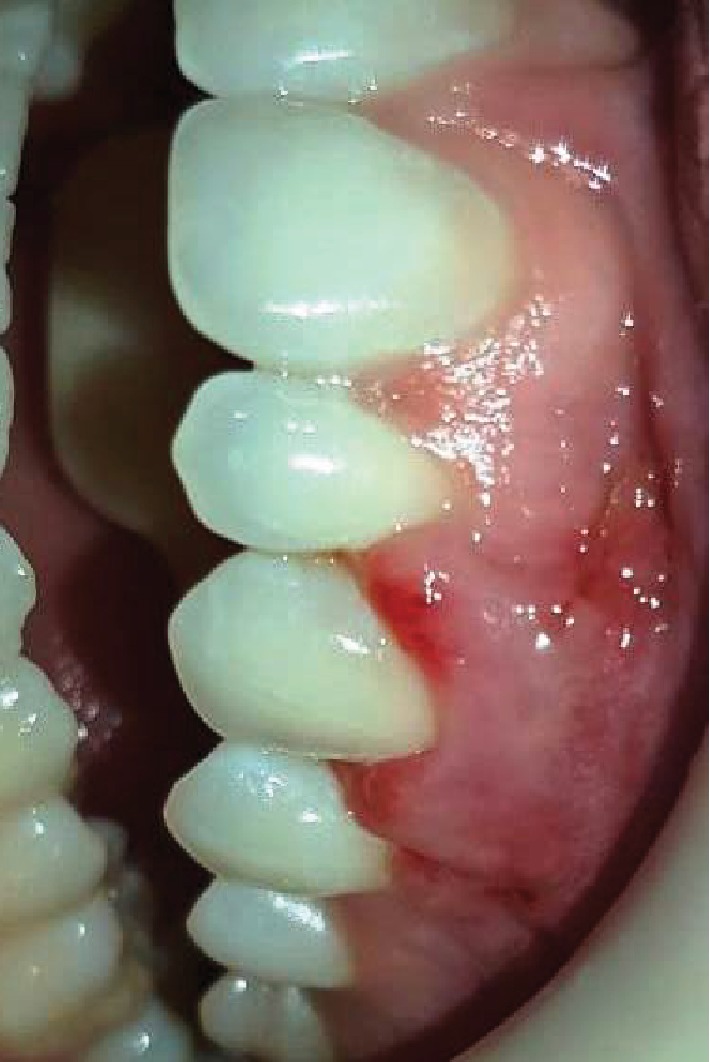
Removal of sutures 15 days postoperatively.

**Figure 12:**
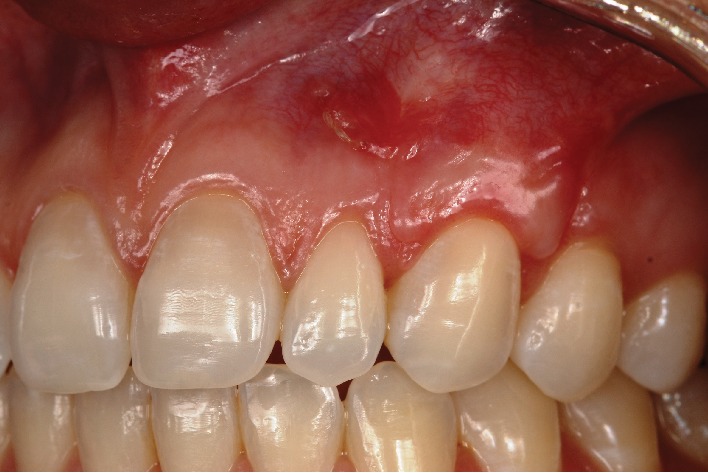
30-day postoperative follow-up.

**Figure 13:**
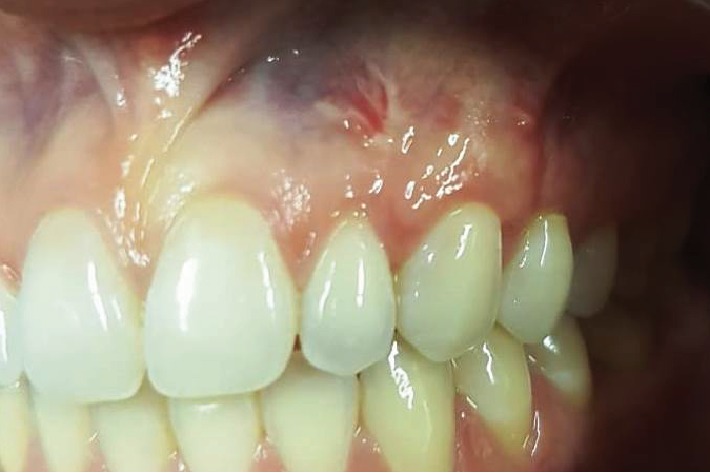
60-day postoperative follow-up.

**Figure 14:**
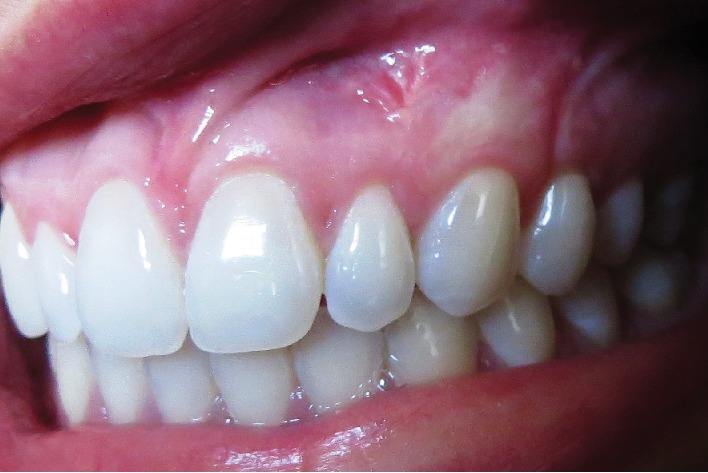
1-year postoperative follow-up.

As postoperative medications, amoxicillin 500 mg (8/8 h for 07 days) and ibuprofen 400 mg (8/8 h for 03 days) were prescribed. The patient was instructed to rinse the surgical site with 0.12% 58 chlorhexidine digluconate solution twice a day, for 1 week. The suture was removed 15 days postsurgery (Figure 11), and follow-up visits were scheduled at 30 (Figure 12), 60 (Figure 13), and 365 (Figure 14) days.

The patient reported that the oral mucosa biting was completely eliminated and that she was satisfied with the esthetic and functional results of the buccal fat pad removal surgery. Additionally, she was satisfied with the root coverage of the maxillary left canine, which favored smile esthetics and reduced dentin sensitivity.

## 3. Discussion

The free gingival graft technique presents good results in the treatment of gingival recessions. However, it is not recommended in esthetic areas, since the color discrepancy is unfavorable [7]. Root coverage using subepithelial connective tissue graft is considered the gold standard when compared to the other surgical techniques, as it provides satisfactory root coverage, gain in clinical attachment level, and keratinized tissue, resulting in therapeutic and esthetic success [10]. However, the palatal donor site usually entails a pronounced postoperative pain, and therefore, different autogenous graft alternatives have been applied in the treatment of gingival recessions [11].

The results of the proposed treatment have been described as positive, and success has been attributed to the ease in harvesting the buccal fat graft through a fast surgical technique with low donor site morbidity and a low rate of complications [12]. The positive results are also attributed to the rich vascular supply that guarantees maintaining the vitality of the graft and the presence of stem cells that aid in tissue regeneration and differentiation of the grafted adipose tissue into stratified squamous epithelium and dense connective tissue [13]. There is a report of long-term maintenance of the graft structure and volume since the buccal fat pad does not undergo lipolysis [14].

El Haddad et al. [7] reported the therapy with pedicled buccal fat pad grafting in a maxillary first molar with the buccal root surface almost completely exposed, presenting grade III mobility and Class II furcation involvement. One month after treatment, there was an average gain of 8 mm in root coverage and gained keratinized tissues with excellent matching of color and texture were observed. In 2010, Kumari et al. [15] used the same technique to cover a Miller Class III defect in a maxillary right molar and obtained a 4 mm gain in the attachment level. Agarwal [6] also applied the technique in a maxillary molar with class IV gingival recession and obtained clinical attachment level and keratinized tissue gain. Ercan et al. [12] described two clinical cases, where the first one showed a gain of 7 mm in the attachment level, reducing the gingival recession to 4 mm. Panda et al. [16] performed a subepithelial graft using the pedicled buccal fat pad and buccal flap advancement for root coverage of a Miller Class III gingival recession exhibiting a 7 mm attachment loss and obtained keratinized tissue gain—which was initially absent—in addition to a gain of 4 mm in the attachment level.

In 2015, Deliberador et al. [8] conducted a split-mouth randomized controlled trial to analyze the transplant efficiency of a nonpedicled buccal fat pad graft for the treatment of Miller Class I and Class II gingival recessions and compared the results with those of a subepithelial connective tissue graft. The authors concluded that the use of a buccal fat pad graft can be considered a predictable alternative, as no significant statistical differences were observed between the two techniques with respect to the esthetic results, gain in clinical attachment level, gain in keratinized tissue, and root coverage; thus, both therapies were considered to be clinically successful.

In the present case report, a nonpedicled buccal fat pad graft was performed with flap advancement, resulting in complete root coverage, with maintenance of the extension of the keratinized tissue width, normal color and texture characteristics, and a small increase in gingival volume. The results were maintained after one year of follow-up suggesting that the technique is safe and effective, in agreement with a previously performed study [8].

## 4. Conclusion

Nonpedicled buccal fat pad graft is an effective technique in the treatment of Miller Class I gingival recessions and may be considered a treatment option. The technique is safe and easy to perform, presents excellent esthetic and therapeutic results, and can also be applied in areas distant from the donor site or even in the lower arch.

## Additional Points

*Key Messages*. The aim of this case report is to address the treatment of a Miller Class I gingival recession using a nonpedicled buccal fat pad graft after buccal fat pad removal surgery (bichectomy technique).
